# Machine learning-based early identification of heart failure/congestion complicating acute coronary syndrome using body composition analysis: a retrospective cohort study

**DOI:** 10.3389/fcvm.2026.1858644

**Published:** 2026-08-24

**Authors:** Yanting Sun, Zhaobin He, Yongzhe Yu

**Affiliations:** 1 Department of Geriatric Medicine, Qilu Hospital of Shandong University, Jinan, Shandong, China; 2 Department of Hepatobiliary Surgery, General Surgery, Qilu Hospital of Shandong University, Jinan, Shandong, China

**Keywords:** acute coronary syndrome, bioelectrical impedance analysis, body composition, heart failure, machine learning

## Abstract

**Background:**

Heart failure (HF) is a major complication of acute coronary syndrome (ACS) and is associated with poor outcomes.

**Aims:**

To develop and internally evaluate a machine learning (ML) model for early identification/risk classification of HF or congestion among patients with ACS using bioelectrical impedance analysis (BIA)-derived body composition parameters.

**Methods:**

This single-center retrospective cohort study included 527 patients with ACS. Body composition was assessed within 48 h of admission using multi-frequency direct segmental BIA. The dataset was first randomly partitioned into training and locked test cohorts. Imputation, standardization, feature selection, and model selection were performed using training data only. A total of 120 model pipelines were compared by training-stage cross-validation, and the locked test set was reserved for one final evaluation.

**Results:**

HF was identified in 198 patients (37.6%). The final plsRglm + glmBoost model used six BIA/body-composition variables: Protein, Muscle—Right arm, Muscle—Trunk, ECW/TBW ratio—Trunk, ECW/TBW ratio—Left leg, and arm muscle circumference. In the locked test set, the model achieved an area under the receiver operating characteristic curve (AUC) of 0.793 (95% CI 0.719–0.859) and a Brier score of 0.186 (95% CI 0.154–0.221). Calibration assessment showed an intercept of 0.266 (95% CI −0.145 to 0.709) and slope of 0.918 (95% CI 0.645–1.287). SHAP analysis indicated that ECW/TBW ratio—Trunk and ECW/TBW ratio—Left leg were the most influential predictors.

**Conclusions:**

A leakage-free ML workflow using bedside BIA-derived body composition parameters showed moderate discrimination for early identification/risk classification of HF/congestion among patients with ACS. Because of the retrospective single-center design, limited timing information, and absence of external validation, the model should not be interpreted as proving prospective prediction of future HF events.

## 1 Introduction

Acute coronary syndrome (ACS) is recognized as one of the primary causes of death across the world (1). ACS is typically classified into three main presentations: unstable angina, non–ST elevation myocardial infarction (NSTEMI), and ST elevation myocardial infarction (STEMI) (2, 3). Patients with ACS are at significant risk of developing heart failure (HF), with clinical trials reporting HF incidence rates from 10.6% to 46% following ACS or myocardial infarction (4–6). Despite improvements, HF mortality rates continue to be high, exceeding those of many cancers. Between 1950 and 1969 and 1990–1999, five-year mortality among men with HF fell from 70% to 59%, while among women it declined from 57% to 45% (7). However, from 2013 to 2017, the decline in mortality rates has decelerated, and HF prevalence and mortality are likely to grow substantially in the next decade (8).

Early identification of ACS patients at high risk of HF is pivotal for tailoring preventive strategies and improving prognosis. Currently, risk stratification largely relies on traditional cardiovascular risk factors, biomarkers (e.g., NT-proBNP), and scoring systems such as the GRACE or TIMI scores (9, 10). However, these models primarily focus on hemodynamic and myocardial injury markers, often overlooking the patient's systemic nutritional and metabolic status. Although body mass index (BMI) is widely used to assess nutritional status, it lacks the sensitivity to distinguish between lean muscle mass and adipose tissue, leading to phenomena such as the “obesity paradox” (11, 12). Consequently, body composition analysis, which quantifies specific compartments such as skeletal muscle mass, visceral fat, and body water, has emerged as a superior prognostic indicator. Emerging evidence suggests that imbalances in body composition—such as sarcopenia or visceral adiposity—are independently associated with adverse cardiovascular outcomes (13, 14).

While body composition analysis offers granular insights into metabolic health, the interplay between various compartments (e.g., muscle-to-fat ratio, phase angle) and HF risk is complex, high-dimensional, and often non-linear. Traditional statistical methods, such as logistic regression or Cox proportional hazards models, may fail to fully capture these intricate interactions, potentially limiting prediction accuracy (15). Machine learning (ML) excels at modeling such non-linear relationships and identifying latent patterns within large clinical datasets. Prior cardiovascular and HF studies have applied ML to predict HF or related outcomes and have suggested potential advantages over conventional statistical approaches in selected settings (16–18). However, the performance of specific ML algorithms (e.g., Random Forest, XGBoost, SVM) varies substantially depending on data structure, and relying on a single algorithm may lead to suboptimal predictions or overfitting (19–21). Consequently, a systematic comparison of multiple ML algorithms is essential. Body composition and fluid-distribution parameters may provide clinically useful bedside signals for identifying HF or congestion during early ACS hospitalization, but their performance should be evaluated with careful attention to overfitting, calibration, and potential information leakage. Therefore, the aim of this study was to develop and internally evaluate a BIA/body-composition-based ML model for early identification and risk classification of HF or congestion among patients with ACS, while systematically comparing multiple model pipelines under a leakage-free workflow.

## 2 Materials and methods

### 2.1 Study design and population

This retrospective cohort study was conducted at the Department of Geriatric Medicine, Qilu Hospital of Shandong University. We retrospectively analyzed the clinical data of patients diagnosed with ACS between January 2024 and December 2025. The diagnosis of ACS, including unstable angina, NSTEMI, and STEMI, was established according to the relevant clinical guidelines.

The study protocol was approved by the Ethics Committee of Qilu Hospital of Shandong University (Approval No. KYLL-202506-006; Date: June 11, 2025) and was conducted in accordance with the Declaration of Helsinki. Due to the retrospective nature of the study, the requirement for informed consent was waived by the Ethics Committee.

Patients were included if they (1) were aged ≥ 18 years, (2) had a confirmed diagnosis of ACS, and (3) underwent body composition analysis within 48 h of admission. The exclusion criteria were: (1) patients with a pacemaker or other implanted electronic devices (contraindication for bioelectrical impedance analysis); (2) patients with severe limb edema or amputation affecting measurement accuracy; (3) patients with end-stage renal disease requiring dialysis; and (4) patients with missing key clinical or laboratory data.

### 2.2 Data collection and body composition analysis

Demographic characteristics, medical history, vital signs, and medication records were extracted from the electronic medical records. Fasting venous blood samples were collected on the morning following admission. Laboratory investigations included complete blood count, coagulation profile, and biochemical indicators (including blood lipids, serum creatinine, and fasting plasma glucose, among others).

Body composition parameters were measured using a portable direct segmental multi-frequency bioelectrical impedance analysis (BIA) device (InBody S10, InBody Co., Ltd., Seoul, Korea). Given the acute condition of the patients, measurements were performed in the supine position by trained nurses. Patients were in a fasting state, with an empty bladder, wearing light clothing, and without metal accessories. Electrodes were attached to the patients' thumbs and middle fingers and to their ankles. Key body composition metrics extracted from the analysis included data from the muscle-fat analysis, segmental lean analysis, segmental body water analysis, and nutritional information [specifically skeletal muscle index [SMI] and visceral fat area [VFA]]. Additionally, the extracellular water-to-total body water ratio (ECW/TBW) was recorded.

### 2.3 Definition of heart failure (outcome label)

The index time was defined as admission/early hospitalization, with BIA performed within 48 h after admission. NT-proBNP measurement and transthoracic echocardiography were obtained during early hospitalization as part of routine clinical assessment. The outcome was defined as HF identified during the index hospitalization.

Patients were classified into the HF group if they had compatible symptoms and/or signs of HF together with elevated NT-proBNP and echocardiographic evidence of cardiac dysfunction. Elevated NT-proBNP was defined using age-specific thresholds (>450 pg/mL for age <50 years, >900 pg/mL for age 50–75 years, and >1,800 pg/mL for age >75 years). Echocardiographic dysfunction included systolic dysfunction or significant diastolic dysfunction. Significant diastolic dysfunction was defined as echocardiographic evidence consistent with elevated left ventricular filling pressure, including abnormal mitral inflow/tissue Doppler indices, increased left atrial volume, or elevated tricuspid regurgitation velocity when available. Where LVEF information was available, HF phenotypes were categorized according to standard LVEF categories; however, phenotype-specific prediction models were not developed because the study endpoint was HF/congestion during the index hospitalization. Patients with isolated transient volume overload or fluid retention without supporting NT-proBNP elevation and echocardiographic cardiac dysfunction were not classified as HF cases. Because exact temporal ordering between BIA measurement and onset of HF manifestations could not always be confirmed retrospectively, the model was interpreted as an early identification/risk classification model for HF/congestion during early hospitalization rather than as a strict prospective model for incident HF after BIA measurement.

### 2.4 Data preprocessing

To minimize information leakage, the dataset was first randomly partitioned into a training set (70%) and a locked test set (30%) using a fixed random seed. All subsequent preprocessing parameters were estimated from the training set only and then applied unchanged to the locked test set. Missingness was low in the dataset. After numeric cleaning, four predictor cells remained missing, involving four patients with at least one missing predictor value; the highest variable-level missingness was 0.569%. Missing values occurred only in inorganic salt (3/527, 0.569%) and ECW/TBW ratio—Left arm (1/527, 0.190%); all other predictors had 0% missingness. Missing continuous predictors were imputed using training-set medians, and missing categorical predictors were imputed using training-set modes. Continuous variables were standardized by z-score normalization using means and standard deviations estimated exclusively from the training set.

Feature ranking and model-pipeline selection were also restricted to the training stage. Candidate BIA/body-composition variables were ranked in the training data according to univariable discrimination and group comparison results, and model-specific variable pre-selection was performed inside the training cross-validation workflow. Within each cross-validation fold, imputation, scaling, variable pre-selection, and model fitting were conducted using the fold-development subset only; the fold-validation subset was used only for internal performance estimation.

### 2.5 Machine learning model development

Twelve distinct machine learning algorithms were systematically evaluated, encompassing linear models, ensemble methods, and probabilistic classifiers. Linear and regularized models included Lasso regression with L1 regularization (*α* = 1), Ridge regression with L2 regularization (*α* = 0), Elastic Net with varying mixing parameters (*α* = 0.1, 0.2, … ,0.9), Stepwise Generalized Linear Model (Stepglm) with forward, backward, and bidirectional selection, Partial Least Squares Regression GLM (plsRglm), and Gradient Boosting Linear Model (glmBoost). Tree-based ensemble methods consisted of Random Forest (RF) using the randomForestSRC package with 1,000 trees, Gradient Boosting Machine (GBM) with 10-fold cross-validation for optimal iteration selection, and Extreme Gradient Boosting (XGBoost) with 5-fold cross-validation. Additional classifiers comprised support vector machine (SVM) with radial basis function kernel, linear discriminant analysis (LDA), and Naïve Bayes classifier.

Given that individual algorithms may exhibit varying performance depending on data characteristics, we implemented a systematic algorithm combination approach. A total of 120 model pipelines, comprising 12 single algorithms and 108 combined models, were evaluated. Combination models used a two-step framework in which the first algorithm performed variable pre-selection and the subsequent algorithm constructed the final predictive model using the selected features. Representative combinations included Lasso + SVM, glmBoost + Ridge, Stepglm + Elastic Net, RF + XGBoost, and plsRglm + glmBoost.

Model-pipeline selection was based on training-stage five-fold cross-validation. The primary selection criterion was cross-validated AUC, with Brier score used as a secondary criterion. To reduce instability and overfitting, eligible final pipelines were required to train successfully across folds and retain no more than six final predictors. The locked test set was not used to choose the final model or tune any preprocessing/modeling decision.

### 2.6 Model evaluation and interpretation

Model performance was assessed using AUC, accuracy (ACC), sensitivity (SEN), specificity (SPE), positive predictive value (PPV), negative predictive value (NPV), F1 score, and Brier score. AUC values were calculated separately for the training and locked test sets using the pROC package. Calibration was evaluated using calibration curves, Brier score, calibration intercept, and calibration slope. Bootstrap confidence intervals were estimated using 2,000 resamples of the fixed final-model predictions; no model refitting or reselection was performed during this bootstrap procedure.

The default classification cutoff was 0.5. In addition, a training-derived Youden cutoff was estimated in the training set and then applied once to the locked test set for supplementary threshold performance. Risk strata were defined using the 33.3rd and 66.7th percentiles of predicted probabilities in the training set. These training-derived thresholds were fixed and then applied to the locked test set.

To enhance model interpretability, SHapley Additive exPlanations (SHAP) analysis was performed for the final selected model (22). SHAP values were calculated for the training cohort (*n* = 368) using the final six model variables and the training data as the background distribution. Because the final model retained six predictors, exact interventional SHAP values were computed on the predicted-probability scale rather than on the log-odds scale. Mean absolute SHAP value was used to rank feature importance. SHAP summary plots, dependence plots, and representative case-level explanations were generated using the final six variables. Dependence plots used original, unstandardized feature values on the *x*-axis, and case-level explanations reported raw feature values to improve clinical interpretability. A reconstruction check confirmed that the sum of the baseline probability and SHAP contributions matched the model-predicted probabilities. SHAP was interpreted as model explanation rather than evidence of causality.

### 2.7 Statistical analysis

All statistical analyses were performed in R (version 4.4.3). Continuous variables are presented as mean (SD) or median (interquartile range), as appropriate, and categorical variables as number (percentage). Between-group comparisons were performed using the Student t test or Mann–Whitney *U*-test for continuous variables and the *χ*^2^-test or Fisher exact test for categorical variables. Two-sided *P* values < 0.05 were considered statistically significant. The train-test split used fixed random seed 42, and subsequent cross-validation/modeling procedures used fixed seeds where applicable. Analyses were run in a reproducible R/pixi workflow, with package/environment information preserved in pixi.toml and pixi.lock. Key R packages included pROC 1.19.0.1, caret 7.0.1, glmnet 5.0, mboost 2.9.11, plsRglm 1.7.1, randomForestSRC 3.6.2, gbm 2.2.3, xgboost 3.3.0.1, e1071 1.7.17, and MASS 7.3.65. The analysis scripts and detailed modeling workflow are available from the corresponding author upon reasonable request.

## 3 Results

**Figure 1:**
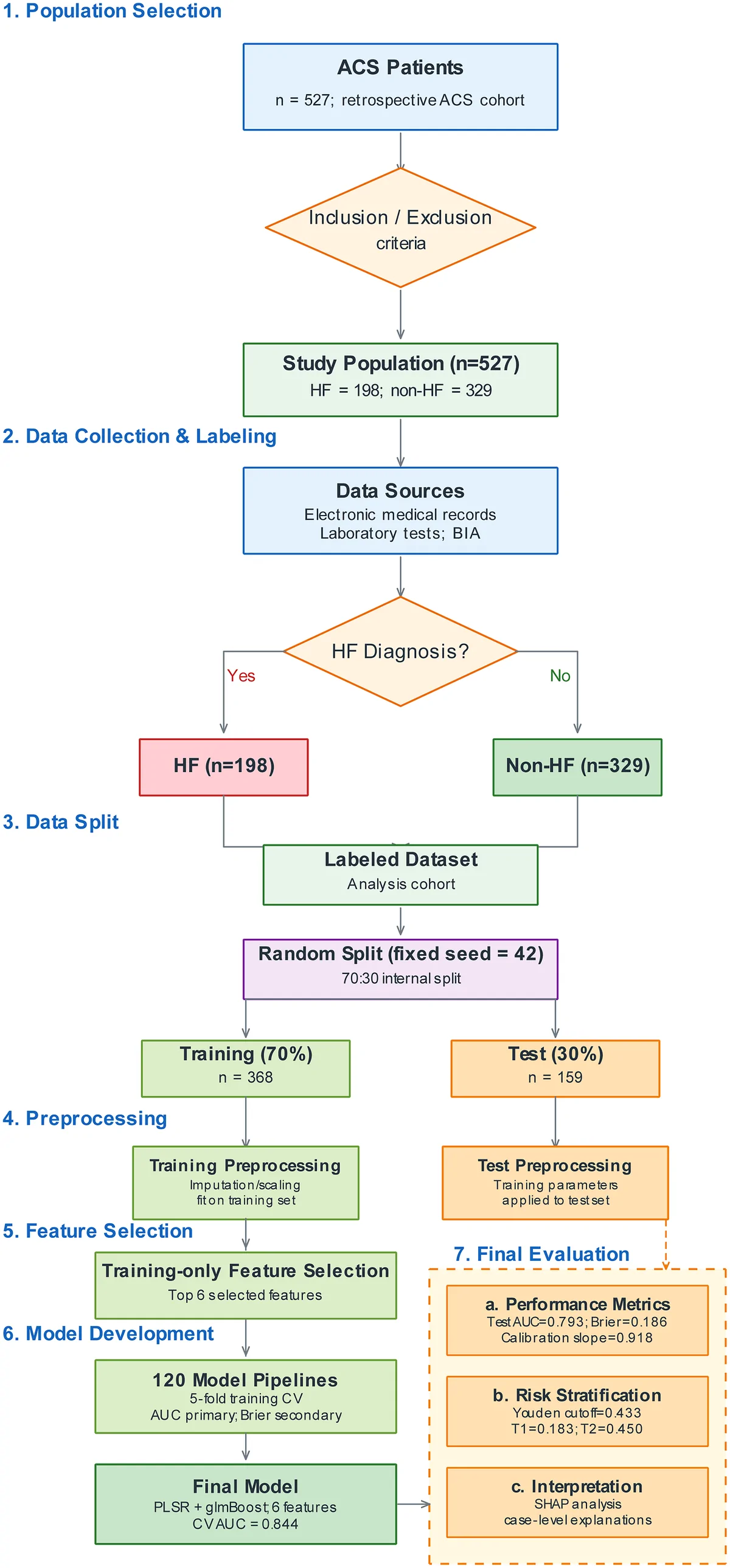
Study workflow diagram. The workflow summarizes cohort construction using the study cohort (*n* = 527), early in-hospital BIA assessment within 48 h of admission, HF/congestion outcome labeling during the index hospitalization, random 70:30 splitting before preprocessing (training *n* = 368; locked test *n* = 159), training-only imputation and standardization, training-only feature selection, training-stage comparison of 120 model pipelines, final-model selection by cross-validation, locked test-set evaluation, training-derived risk stratification, and SHAP-based interpretation. ACS, acute coronary syndrome; BIA, bioelectrical impedance analysis; HF, heart failure; CV, cross-validation; AUC, area under the receiver operating characteristic curve; SHAP, SHapley Additive exPlanations.

The overall study workflow is illustrated in Figure 1, encompassing population selection, early in-hospital BIA assessment, outcome labeling, train-test splitting before preprocessing, training-only imputation/scaling, training-stage model selection, locked test-set evaluation, and model interpretation.

### 3.1 Baseline characteristics of study population

A total of 527 patients with ACS who met the inclusion criteria were included in the analysis. HF/congestion was identified in 198 patients (37.6%), while 329 patients (62.4%) were classified into the Non-HF group. The baseline demographic characteristics, laboratory parameters, and body composition measurements are summarized in Table 1. Age, sex, smoking history, drinking history, hypertension, diabetes, height, weight, and BMI were not significantly different between groups. Several BIA/body-composition measures differed between the groups, including Protein, fat mass, fat percentage, Muscle—Right arm, Muscle—Trunk, ECW/TBW ratio—Trunk, ECW/TBW ratio—Left leg, and arm muscle circumference. The ECW/TBW ratio—Left leg was 0.38 (0.38, 0.39) in the Non-HF group and 0.39 (0.38, 0.40) in the HF group (*p* < 0.001).

**Table 1:** Baseline characteristics of the study cohort.

Characteristic	Non-HF (*n* = 329)	HF (*n* = 198)	p
Gender, *n* (%)			
Female	105 (31.91)	53 (26.77)	0.212
Male	224 (68.09)	145 (73.23)	
Smoke history, *n* (%)			
Nonsmoker	179 (54.41)	99 (50.00)	0.326
Smoker	150 (45.59)	99 (50.00)	
Drinking history, *n* (%)			
Nondrinker	184 (55.93)	115 (58.08)	0.629
Drinker	145 (44.07)	83 (41.92)	
Hypertension, *n* (%)			
No	109 (33.13)	66 (33.33)	0.962
Yes	220 (66.87)	132 (66.67)	
Diabetes, *n* (%)			
No	224 (68.09)	129 (65.15)	0.488
Yes	105 (31.91)	69 (34.85)	
Age, median (IQR)	60.00 (54.00, 68.00)	63.00 (54.00, 69.00)	0.330
Height, median (IQR)	168.00 (162.00, 173.00)	168.00 (162.00, 172.00)	0.761
Weight, median (IQR)	72.00 (63.00, 82.00)	72.00 (63.00, 80.00)	0.623
BMI, median (IQR)	25.70 (23.30, 28.60)	25.50 (23.50, 28.00)	0.462
ICW, median (IQR)	24.70 (20.50, 28.30)	24.70 (20.90, 28.10)	0.638
ECW, median (IQR)	14.90 (12.50, 17.10)	14.95 (13.00, 17.00)	0.470
ECW/TBW ratio, median (IQR)	0.38 (0.37, 0.39)	0.38 (0.37, 0.39)	0.585
TBW/FFM, median (IQR)	73.40 (73.20, 73.60)	73.40 (73.20, 73.70)	0.414
Protein, median (IQR)	11.00 (10.10, 11.90)	10.35 (9.70, 11.00)	<0.001
Inorganic salt, median (IQR)	3.66 (3.14, 4.27)	3.66 (3.26, 4.31)	0.498
Fat mass, median (IQR)	19.60 (13.90, 24.50)	18.15 (13.20, 22.70)	0.041
Fat percentage, median (IQR)	26.70 (21.30, 31.80)	24.70 (19.10, 30.20)	0.045
VFA, median (IQR)	79.30 (53.50, 101.20)	71.20 (45.30, 99.10)	0.075
Muscle mass, median (IQR)	30.20 (24.70, 34.90)	30.20 (25.30, 34.60)	0.646
Muscle—Right arm, median (IQR)	2.95 (2.65, 3.29)	2.57 (2.28, 2.94)	<0.001
Muscle—Left arm, median (IQR)	2.74 (2.12, 3.21)	2.75 (2.18, 3.21)	0.808
Muscle—Trunk, median (IQR)	23.60 (21.61, 25.62)	22.06 (20.19, 23.75)	<0.001
Muscle—Right leg, median (IQR)	8.94 (7.40, 10.50)	9.17 (7.68, 10.29)	0.495
Muscle—Left leg, median (IQR)	8.78 (7.29, 10.22)	8.96 (7.50, 10.15)	0.496
Water—Right arm, median (IQR)	2.15 (1.67, 2.55)	2.16 (1.71, 2.52)	0.916
Water—Left arm, median (IQR)	2.14 (1.65, 2.49)	2.14 (1.69, 2.49)	0.790
Water—Trunk, median (IQR)	17.80 (14.90, 20.10)	17.80 (15.20, 19.90)	0.986
Water—Right leg, median (IQR)	6.97 (5.79, 8.18)	7.17 (5.97, 7.95)	0.472
Water—Left leg, median (IQR)	6.85 (5.68, 7.97)	6.97 (5.86, 7.85)	0.547
ECW/TBW ratio—Right arm, median (IQR)	0.38 (0.37, 0.38)	0.38 (0.37, 0.38)	0.286
ECW/TBW ratio—Left arm, median (IQR)	0.38 (0.38, 0.38)	0.38 (0.38, 0.38)	0.095
ECW/TBW ratio—Trunk, median (IQR)	0.38 (0.37, 0.38)	0.38 (0.38, 0.39)	<0.001
ECW/TBW ratio—Right leg, median (IQR)	0.38 (0.37, 0.39)	0.38 (0.37, 0.39)	0.614
ECW/TBW ratio—Left leg, median (IQR)	0.38 (0.38, 0.39)	0.39 (0.38, 0.40)	<0.001
BCM, median (IQR)	35.40 (29.10, 40.50)	35.35 (29.80, 40.20)	0.590
BMC, median (IQR)	3.02 (2.60, 3.51)	3.01 (2.65, 3.57)	0.445
Arm circumference, median (IQR)	30.80 (28.70, 33.40)	30.60 (28.70, 32.80)	0.284
Arm muscle circumference, median (IQR)	27.40 (26.30, 28.80)	26.00 (24.70, 27.30)	<0.001
Waist circumference, median (IQR)	83.00 (76.60, 91.60)	80.80 (75.10, 90.60)	0.141
BMR, median (IQR)	1,548.00 (1,337.00, 1,714.00)	1,545.00 (1,368.00, 1,693.00)	0.624
SMI, median (IQR)	8.30 (7.20, 9.10)	8.30 (7.40, 9.20)	0.448

Values are presented as median (IQR) for continuous variables and *n* (%) for categorical variables.

HF, heart failure; BMI, body mass index; ICW, intracellular water; ECW, extracellular water; TBW, total body water; FFM, fat-free mass; VFA, visceral fat area; BCM, body cell mass; BMC, bone mineral content; BMR, basal metabolic rate; SMI, skeletal muscle index.

### 3.2 Feature selection results

Training-only feature ranking identified six final BIA/body-composition variables for the selected model: Protein, Muscle—Right arm, Muscle—Trunk, ECW/TBW ratio—Trunk, ECW/TBW ratio—Left leg, and arm muscle circumference (AMC). ECW/TBW ratio—Trunk and ECW/TBW ratio—Left leg had higher values in the HF group, whereas Protein, Muscle—Right arm, Muscle—Trunk, and AMC tended to be lower in the HF group. These variables were retained in the final plsRglm + glmBoost model selected during training-stage cross-validation.

### 3.3 Machine learning model performance

**Figure 2:**
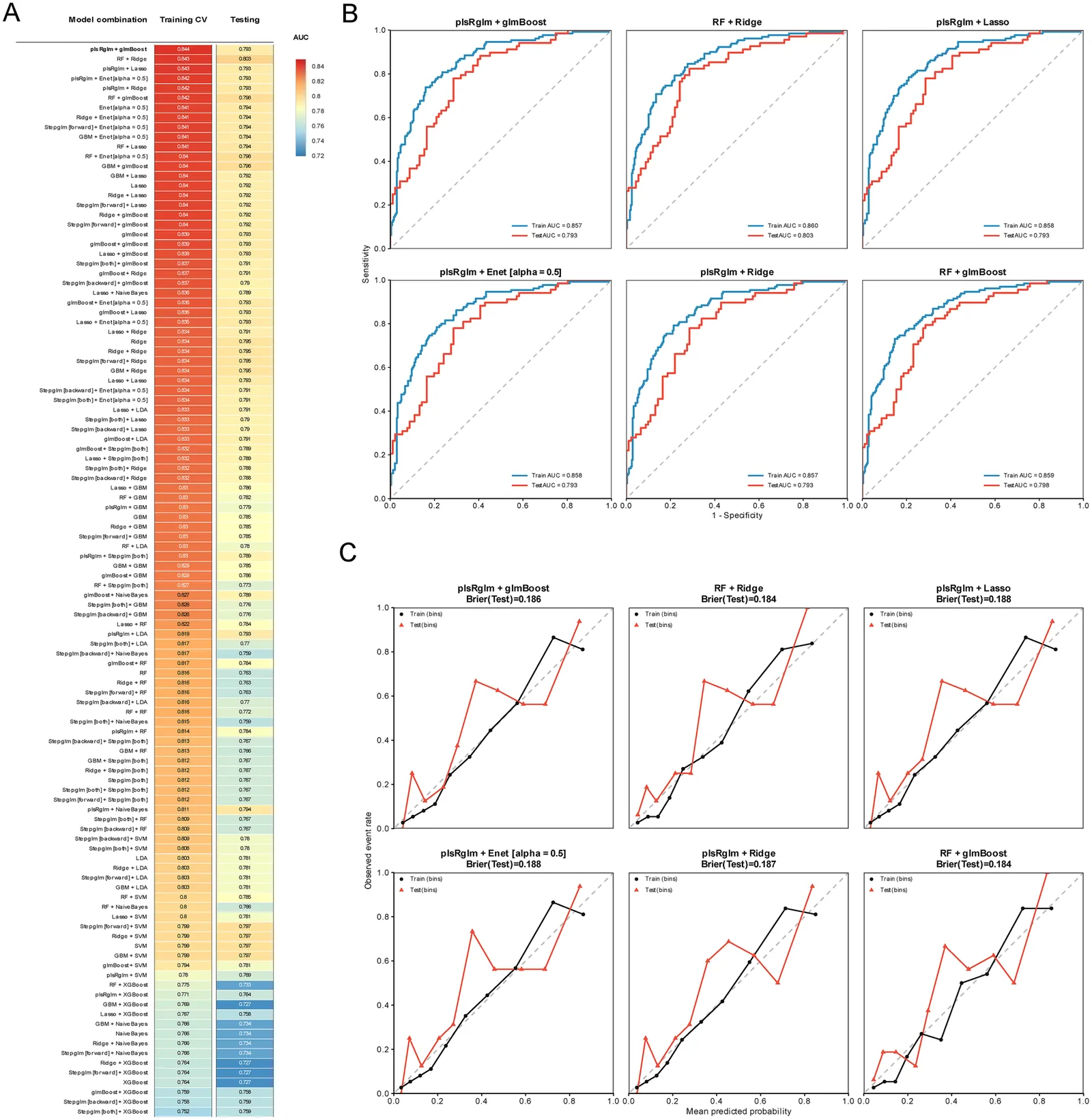
Machine learning model-pipeline comparison and final-model selection. **(A)** A total of 120 model pipelines were attempted during training-stage cross-validation; the heatmap displays pipelines with valid performance estimates after excluding unsuccessful/NA configurations, with the selected final model highlighted. **(B)** Receiver operating characteristic curves for the top six training-CV-ranked model pipelines in the training and locked test cohorts. Test-set AUCs were reported only after model selection and were not used to choose the final model. **(C)** Calibration curves for the top six model pipelines. AUC, area under the receiver operating characteristic curve; CV, cross-validation; RF, random forest.

A total of 120 model pipelines were compared during training-stage five-fold cross-validation. Figure 2A shows the model-pipeline heatmap. The final model was selected according to training cross-validated AUC, with Brier score used as a secondary criterion; the locked test set was not used for model selection. The selected final pipeline was plsRglm + glmBoost, with a training cross-validated AUC of 0.844.

After refitting the selected model in the full training cohort, the training AUC was 0.857 and the locked test AUC was 0.793. In the locked test set, the final model achieved accuracy 0.704, sensitivity 0.529, specificity 0.835, PPV 0.706, NPV 0.704, F1 score 0.605, and Brier score 0.186 using the default 0.5 cutoff (Table 2). Bootstrap analysis of the fixed final predictions showed a test AUC of 0.793 (95% CI 0.719–0.859) and Brier score of 0.186 (95% CI 0.154–0.221).

**Table 2:** Performance metrics of the top six model pipelines in the training and locked test cohorts.

Model	Dataset	AUC	ACC	SEN	SPE	PPV	NPV	F1	Brier
plsRglm + glmBoost	Train	0.857	0.796	0.615	0.895	0.762	0.810	0.681	0.145
plsRglm + glmBoost	Test	0.793	0.704	0.529	0.835	0.706	0.704	0.605	0.186
RF + Ridge	Train	0.860	0.796	0.608	0.899	0.767	0.808	0.678	0.144
RF + Ridge	Test	0.803	0.704	0.515	0.846	0.714	0.700	0.598	0.184
plsRglm + Lasso	Train	0.858	0.791	0.600	0.895	0.757	0.804	0.670	0.144
plsRglm + Lasso	Test	0.793	0.704	0.529	0.835	0.706	0.704	0.605	0.188
plsRglm + Enet[alpha=0.5]	Train	0.858	0.791	0.600	0.895	0.757	0.804	0.670	0.144
plsRglm + Enet[alpha=0.5]	Test	0.793	0.698	0.515	0.835	0.700	0.697	0.593	0.188
plsRglm + Ridge	Train	0.857	0.788	0.600	0.891	0.750	0.803	0.667	0.145
plsRglm + Ridge	Test	0.793	0.698	0.515	0.835	0.700	0.697	0.593	0.187
RF + glmBoost	Train	0.859	0.791	0.615	0.887	0.748	0.808	0.675	0.145
RF + glmBoost	Test	0.798	0.698	0.529	0.824	0.692	0.701	0.600	0.184

The six model pipelines are ordered according to training-stage cross-validated AUC. Test-set metrics were calculated once in the locked test cohort and were not used for model selection.

AUC, area under the receiver operating characteristic curve; ACC, accuracy; SEN, sensitivity; SPE, specificity; PPV, positive predictive value; NPV, negative predictive value; F1, F1 score; Brier, Brier score; CV, cross-validation.

The six model pipelines shown in Figure 2B and Table 2 were ordered by training-stage cross-validated performance. Although RF + Ridge showed a slightly higher locked test AUC (0.803) than the selected final model, it was not selected retrospectively because using test-set performance for model choice would violate the locked-test-set evaluation strategy. Therefore, test-set AUCs in Figure 2B are reported only as final held-out evaluations after model selection.

### 3.4 Model calibration and clinical utility

Calibration curves for the top six model pipelines are shown in Figure 2C. For the final plsRglm + glmBoost model, the locked test-set calibration intercept was 0.266 (95% CI −0.145 to 0.709), and the calibration slope was 0.918 (95% CI 0.645–1.287). These results suggested acceptable but uncertain calibration in the locked test set.

**Figure 3:**
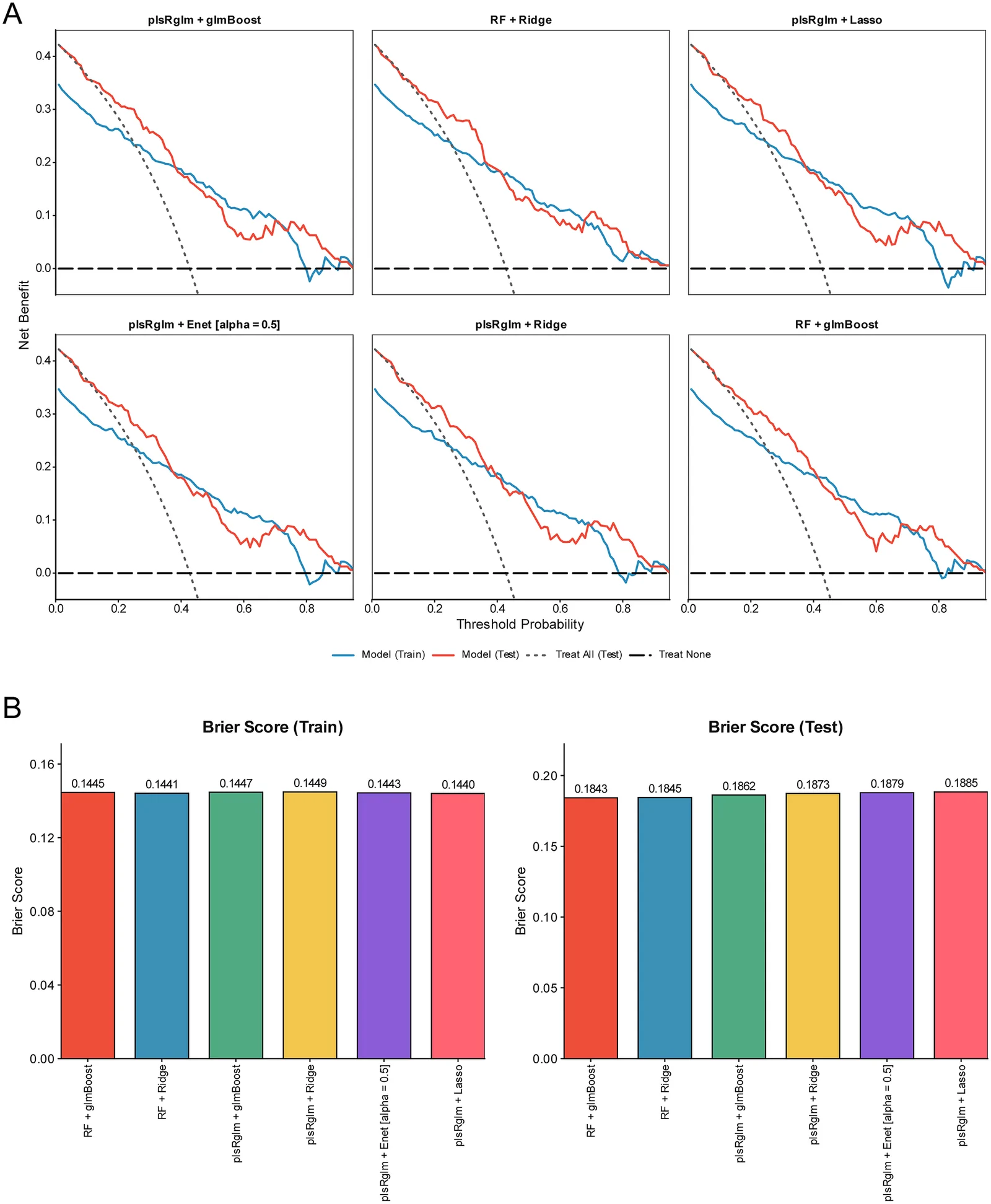
Clinical utility and prediction-error assessment. **(A)** Decision curve analysis for the top six model pipelines, interpreted in the context of early HF/congestion risk classification and prioritization for closer monitoring or further assessment rather than automatic treatment decisions. **(B)** Brier score comparison across the top six model pipelines in the training and locked test cohorts. DCA, decision curve analysis; HF, heart failure.

DCA was performed to explore potential net benefit for early HF/congestion risk classification and prioritization for closer monitoring or further assessment, rather than automatic treatment decisions (Figure 3A). A training-derived Youden cutoff of 0.433 was applied once to the locked test set for supplementary classification assessment. At this cutoff, test sensitivity was 0.603 (95% CI 0.483–0.723), specificity was 0.780 (95% CI 0.690–0.861), and accuracy was 0.704 (Table 3). Brier score comparisons across the top six model pipelines are shown in Figure 3B.

**Table 3:** Risk stratification based on predicted probability from the final plsRglm + glmBoost model.

Part 1. Risk strata in the locked test cohort						
Risk group	Probability range	n	Observed HF incidence, % (95% CI)			
Low	*p* < 0.183	44	11.4% (5/44), 3.8–24.6			
Intermediate	0.183 ≤ *p* < 0.450	56	41.1% (23/56), 28.1–55.0			
High	*p* ≥ 0.450	59	67.8% (40/59), 54.4–79.4			

Part 2. Threshold performance in the locked test cohort.						
Threshold evaluated	Classification rule	TP/FP/TN/FN	Sensitivity	Specificity	PPV	NPV
Default cutoff = 0.500	Predicted HF if *p* ≥ 0.500	36/15/76/32	52.9%	83.5%	70.6%	70.4%
Training Youden cutoff = 0.433	Predicted HF if *p* ≥ 0.433	41/20/71/27	60.3%	78.0%	67.2%	72.4%
T_low = 0.183	Predicted HF if *p* ≥ 0.183	63/52/39/5	92.6%	42.9%	54.8%	88.6%
T_high = 0.450	Predicted HF if *p* ≥ 0.450	40/19/72/28	58.8%	79.1%	67.8%	72.0%

Risk strata were defined using the 33.3rd and 66.7th percentiles of model-predicted probabilities in the training set (T_low = 0.183 and T_high = 0.450). These training-derived thresholds were fixed and then applied to the locked test cohort.

HF, heart failure; NPV, negative predictive value; PPV, positive predictive value; TP, true positives; FP, false positives; TN, true negatives; FN, false negatives.

**Figure 4:**
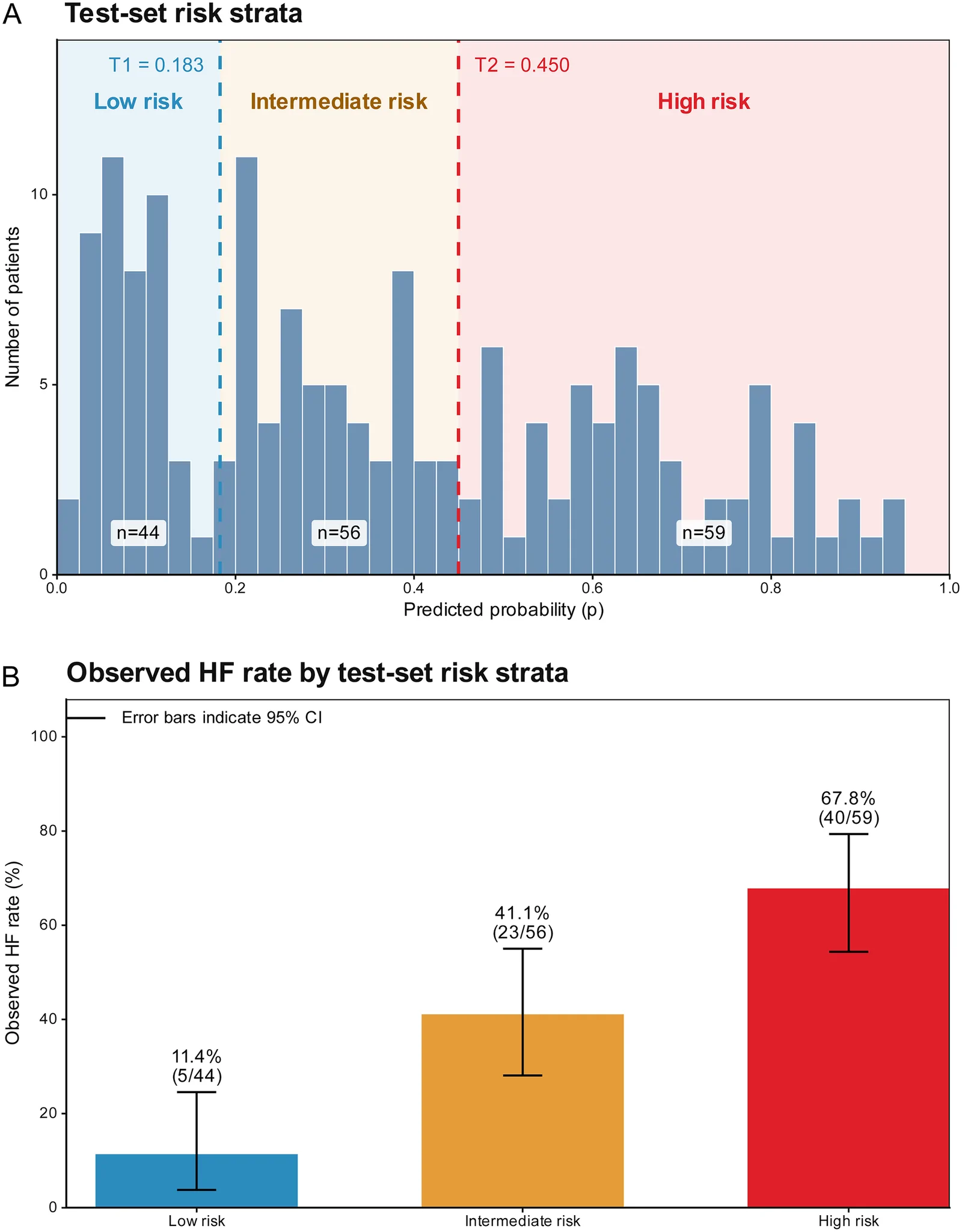
Training-derived risk stratification using the final plsRglm + glmBoost model. **(A)** Predicted probability distribution in the locked test set using training-derived tertile thresholds (T1/T_low = 0.183 and T2/T_high = 0.450). **(B)** Observed HF/congestion incidence across low-, intermediate-, and high-risk strata in the locked test set with 95% confidence intervals.

Risk strata were derived from the 33.3rd and 66.7th percentiles of training-set predicted probabilities (T_low = 0.183 and T_high = 0.450) and then applied to the locked test cohort (Figure 4 and Table 3). In the locked test set, observed HF incidence was 11.4% (5/44) in the low-risk group, 41.1% (23/56) in the intermediate-risk group, and 67.8% (40/59) in the high-risk group. These strata should be interpreted as exploratory training-derived risk categories rather than prespecified clinical treatment thresholds.

### 3.5 SHAP-based model interpretation

**Figure 5:**
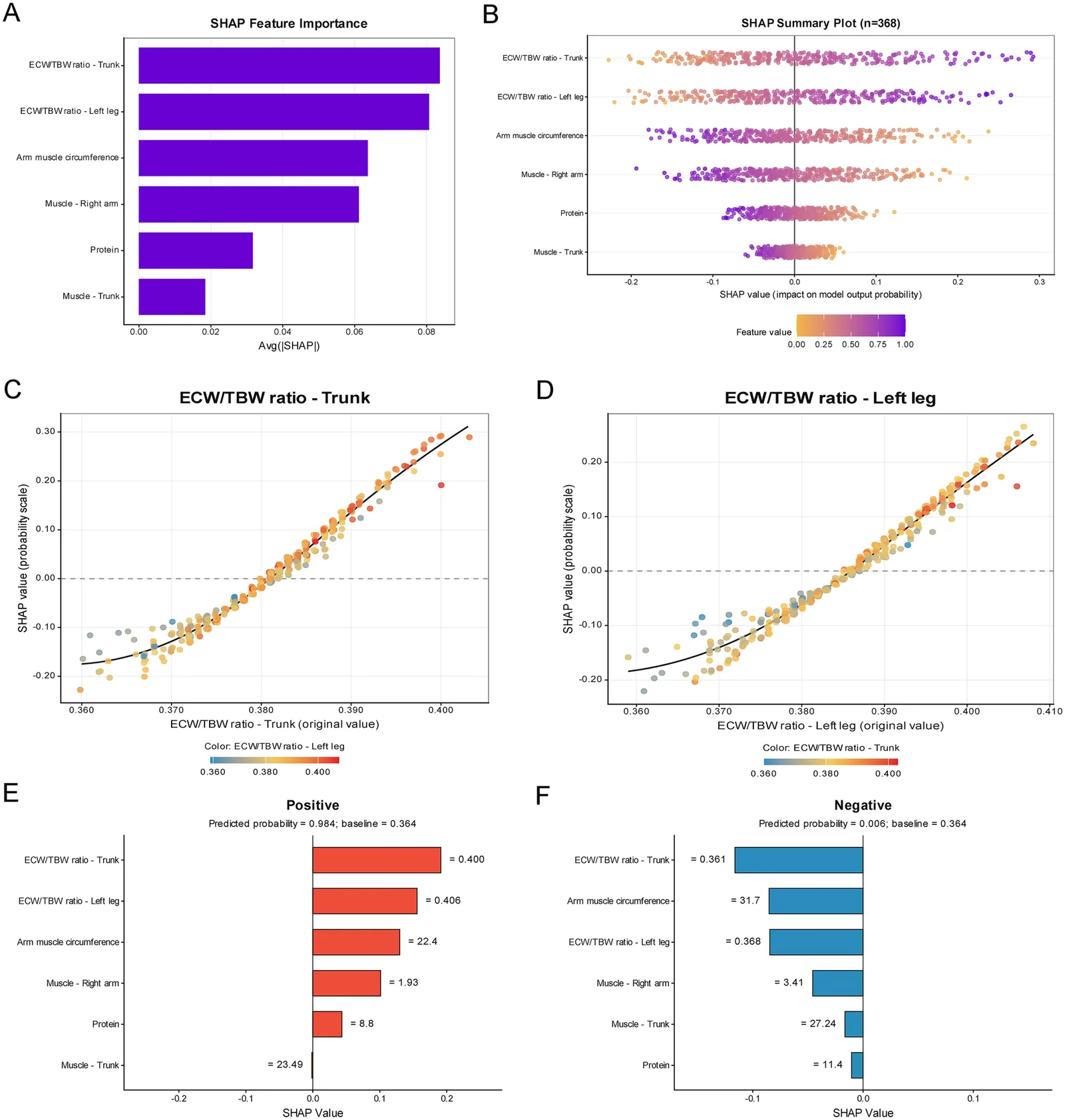
SHAP analysis for interpretation of the final plsRglm + glmBoost model. **(A)** Feature importance ranking based on mean absolute SHAP values. SHAP values were computed in the training cohort (*n* = 368) using the training data as the background distribution and model outputs on the probability scale. **(B)** SHAP summary plot for the final six model variables. **(C)** SHAP dependence plot for ECW/TBW ratio—Trunk using original feature values, colored by ECW/TBW ratio—Left leg. **(D)** SHAP dependence plot for ECW/TBW ratio—Left leg using original feature values, colored by ECW/TBW ratio—Trunk. **(E)** Case-level SHAP explanation for a representative positive/high-probability prediction with raw feature values. **(F)** Case-level SHAP explanation for a representative negative/low-probability prediction with raw feature values. SHAP values indicate model contribution and do not establish causality. SHAP, SHapley Additive exPlanations; ECW/TBW, extracellular water to total body water ratio; AMC, arm muscle circumference; HF, heart failure.

To enhance model interpretability, SHAP analysis was performed for the final selected model using the final six variables (Figure 5). The feature-importance ranking based on mean absolute SHAP values identified ECW/TBW ratio—Trunk and ECW/TBW ratio—Left leg as the two most influential features, followed by AMC, Muscle—Right arm, Protein, and Muscle—Trunk (Figure 5A). SHAP summary and dependence plots were generated on the probability scale, and dependence plots used original unstandardized feature values.

Higher ECW/TBW ratio—Trunk and ECW/TBW ratio—Left leg values generally contributed toward higher model-predicted probability of HF/congestion (Figures 5B–D). Muscle/nutritional variables contributed more modestly and should be interpreted as part of the multivariable model rather than as independent causal mechanisms. Representative case explanations (Figures 5E,F) illustrate how the final model combined fluid-distribution and muscle/nutritional variables to generate individual predictions. These SHAP findings were interpreted as model-explanation results and not as evidence of causal pathophysiological effects.

## 4 Discussion

In this study, we developed and internally evaluated a BIA/body-composition-based ML model for early identification and risk classification of HF/congestion among patients with ACS. The final plsRglm + glmBoost model, selected by training-stage cross-validation, achieved moderate and clinically plausible performance in the locked test set, with an AUC of 0.793 and a Brier score of 0.186.

The results support the presence of an interpretable BIA/body-composition signal. The final model showed moderate discrimination, and the default 0.5 cutoff showed limited sensitivity. A training-derived Youden cutoff improved test sensitivity at the cost of lower specificity, which may be more compatible with early screening or prioritization for further HF assessment. These findings suggest that the model may help identify patients who warrant closer monitoring or additional evaluation, but they do not establish that model-guided intervention improves clinical outcomes.

Fluid-distribution variables remained important in the final model. ECW/TBW ratio reflects the proportion of extracellular water relative to total body water and is commonly interpreted as an indicator of fluid overload or tissue edema (23–27). Previous studies have reported associations between ECW/TBW, natriuretic peptide levels, congestion, and outcomes in patients with HF or dyspnea (23–26). In our SHAP analysis, ECW/TBW ratio—Trunk and ECW/TBW ratio—Left leg were the two most influential model features. This pattern is clinically plausible because systemic and segmental fluid redistribution may reflect early congestion. However, this finding must be interpreted cautiously because the HF outcome definition also included clinical signs/symptoms, which have limited diagnostic specificity when used in isolation (28), and NT-proBNP elevation. Therefore, BIA-derived ECW/TBW variables may partly capture the same congestion state used to define HF. We consequently frame the model as an early identification/risk classification tool for HF/congestion during early hospitalization, rather than as proof of strict temporal prediction of future incident HF.

The final model also included muscle and nutritional variables, including Muscle—Right arm, Muscle—Trunk, Protein, and AMC. These features are consistent with literature linking sarcopenia, muscle depletion, and poor nutritional status with adverse cardiovascular outcomes (29–35). In the study cohort, Protein, arm muscle circumference, and selected segmental muscle variables were lower in the HF group. These variables may reflect frailty, catabolic state, or baseline vulnerability, although causal interpretation is not warranted from this retrospective analysis. Nutritional assessment and nutritional interventions have also been discussed in patients with chronic HF and cardiopulmonary disease, but our study did not evaluate treatment effects (36, 37). Their inclusion suggests that body composition may provide complementary physiologic context beyond a single fluid-overload marker.

A key methodological feature of this study was the separation of model selection from final test-set evaluation. The 120 pipelines were compared using training-stage cross-validation, and the locked test set was evaluated once after the final pipeline had been selected. This approach avoids choosing a model because it happened to perform best in the test set. For example, RF + Ridge showed a slightly higher locked test AUC than the selected final model, but it was not chosen retrospectively because doing so would have converted the test set into a model-selection set. This distinction is important for transparent reporting of ML prediction models and directly addresses the risk of optimistic performance estimation.

SHAP analysis was used to improve interpretability of the final model. Explainability is increasingly emphasized as a prerequisite for clinical translation of ML models, but explanation methods must still be interpreted cautiously (38, 39). The SHAP plots were generated for the final plsRglm + glmBoost model and final six variables, with dependence plots using original feature values and case-level explanations reporting raw feature values. SHAP values should be interpreted as model contributions on the prediction scale, not as evidence of causal effects or biological mechanisms. Accordingly, the SHAP findings are hypothesis-generating and should be confirmed in external datasets.

The findings of this study have several potential clinical implications. BIA provides rapid, noninvasive bedside measurements and may help identify ACS patients who warrant closer monitoring, repeat biomarker testing, echocardiographic evaluation, or more careful assessment of volume status. However, the present model should not be used to trigger automatic treatment decisions. Decision-curve analysis was interpreted only in the context of early risk classification and prioritization for further HF/congestion assessment. Prospective evaluation is required before model-guided interventions can be recommended.

This study has several limitations. First, it was a single-center retrospective study, and no independent external cohort was available. External validation in multicenter and preferably prospective cohorts is required before clinical implementation. Second, the timing of BIA measurement, NT-proBNP testing, echocardiography, and onset of HF manifestations could not always be precisely ordered. Therefore, the model may classify existing or early manifest HF/congestion rather than predict strictly future incident HF. Third, there is potential circularity between BIA-derived fluid-overload variables and the HF outcome definition, which incorporated clinical congestion and NT-proBNP. Fourth, several conventional clinical predictors, including Killip class, LVEF, troponin, renal function, ACS subtype, prior HF, revascularization strategy, medication use, and GRACE/TIMI scores, were not uniformly available in the current analysis dataset. Thus, we could not construct a complete conventional clinical-risk comparator model, and the present study does not prove incremental value beyond established clinical predictors. Fifth, the HF prevalence remained relatively high at 37.6%, which may reflect patient selection, the single-center geriatric medicine setting, and the retrospective outcome definition. Sixth, BIA measurements can be influenced by hydration status, food intake, and body position, although standardized procedures were used to reduce measurement variability (27, 40). Finally, bootstrap confidence intervals quantify uncertainty in the fixed final predictions and do not fully account for model-selection uncertainty.

## 5 Conclusions

In conclusion, a leakage-free ML workflow using BIA-derived body composition parameters showed moderate discrimination for early identification/risk classification of HF/congestion among patients with ACS. The final plsRglm + glmBoost model selected by training-stage cross-validation achieved a locked test AUC of 0.793, with ECW/TBW ratio—Trunk, ECW/TBW ratio—Left leg, and muscle/nutritional variables contributing to model predictions. These findings support further study of bedside BIA as an exploratory tool for early HF/congestion assessment, but they do not establish prospective prediction of future HF or incremental value beyond established clinical risk models. Future prospective multicenter studies with external validation and complete clinical covariates are needed before clinical implementation.

## Data Availability

The de-identified data supporting the findings of this study and the analysis code and modeling workflow are available from the corresponding author upon reasonable request, subject to institutional and ethical approval requirements.
